# Functional and Biochemical Characterization of *Alvinella pompejana* Cys-Loop Receptor Homologues

**DOI:** 10.1371/journal.pone.0151183

**Published:** 2016-03-21

**Authors:** Eveline Wijckmans, Mieke Nys, Sarah Debaveye, Marijke Brams, Els Pardon, Katrien Willegems, Daniel Bertrand, Jan Steyaert, Rouslan Efremov, Chris Ulens

**Affiliations:** 1 Department of Cellular and Molecular Medicine, Laboratory of Structural Neurobiology, University of Leuven, Leuven, Belgium; 2 Structural Biology Brussels, Vrije Universiteit Brussel, Brussels, Belgium; 3 Structural Biology Research Center, VIB, Brussels, Belgium; 4 HiQscreen, Geneva, Switzerland; University of Roskilde, DENMARK

## Abstract

Cys-loop receptors are membrane spanning ligand-gated ion channels involved in fast excitatory and inhibitory neurotransmission. Three-dimensional structures of these ion channels, determined by X-ray crystallography or electron microscopy, have revealed valuable information regarding the molecular mechanisms underlying ligand recognition, channel gating and ion conductance. To extend and validate the current insights, we here present promising candidates for further structural studies. We report the biochemical and functional characterization of Cys-loop receptor homologues identified in the proteome of *Alvinella pompejana*, an extremophilic, polychaete annelid found in hydrothermal vents at the bottom of the Pacific Ocean. Seven homologues were selected, named *Alpo*1-*7*. Five of them, *Alpo*2-6, were unidentified prior to this study. Two-electrode voltage clamp experiments revealed that wild type *Alpo*5 and *Alpo*6, both sharing remarkably high sequence identity with human glycine receptor α subunits, are anion-selective channels that can be activated by glycine, GABA and taurine. Furthermore, upon expression in insect cells fluorescence size-exclusion chromatography experiments indicated that four homologues, *Alpo*1, *Alpo*4, *Alpo*6 and *Alpo*7, can be extracted out of the membrane by a wide variety of detergents while maintaining their oligomeric state. Finally, large-scale purification efforts of *Alpo*1, *Alpo*4 and *Alpo*6 resulted in milligram amounts of biochemically stable and monodisperse protein. Overall, our results establish the evolutionary conservation of glycine receptors in annelids and pave the way for future structural studies.

## Introduction

Cys-loop receptors (CLRs) or pentameric ligand-gated ion channels (pLGICs) are a class of integral membrane proteins located in the central and peripheral nervous system. They mediate fast synaptic neurotransmission by opening up their ion channel pore upon allosteric binding of neurotransmitters to the extracellular ligand binding domain. This results in ion flux, which depolarizes or hyperpolarizes the postsynaptic cell. Because of their pivotal role in neurotransmission, dysfunction of these receptors is linked to several neurological disorders including Alzheimer’s disease [1], epilepsy [2–4], myasthenia gravis [5] and hyperekplexia [6]. Additionally, they are the target of several clinically important drugs like benzodiazepines, general anesthetics, anti-emetics and smoking cessation aids. Due to this high clinical relevance, detailed structural insights about these receptors might assist in unraveling the molecular mechanisms of diseases correlated to CLRs. Moreover, this structural information can lead to novel pathways for the development of more efficient drugs.

Over the last decade, several X-ray crystal structures have been solved for this class of ion channels. Crystal structures of Acetylcholine Binding Proteins (AChBPs), water-soluble homologues of the extracellular domain of nicotinic acetylcholine receptors (nAChRs), in complex with a wide variety of known CLR ligands have provided insights concerning the molecular determinants of ligand recognition [7]. On the other hand, structures of the full-length prokaryotic channels ELIC [8] and GLIC [9–11] have offered a first glimpse of channel gating and ion conductance. ELIC is derived from the bacterium *Erwinia chrysanthemi* and can be activated by primary amines, such as GABA [12,13]. So far its structure was only solved in a non-conductive conformation [8]. By contrast, X-ray crystal structures of GLIC, derived from *Gloeobacter violaceus*, revealed both open and closed conformations [9–11]. Similarly, X-ray crystal structures of GluCl, a glutamate-gated chloride channel derived from *Caenorhabditis elegans*, were determined in different conformational states [14,15]. In 2014, structural information of vertebrate Cys-loop receptors emerged from the elucidation of the human GABA β3 receptor (hGABA_A_R β3) [16] and the mouse 5-HT_3_A receptor (m5-HT_3_A R) [17] structures. More recently, structures became available of the zebrafish glycine α1 receptor (zGlyR α1) and of the human glycine α3 receptor (hGlyR α3), offering further insights about channel gating [18,19].

**Fig 1:**
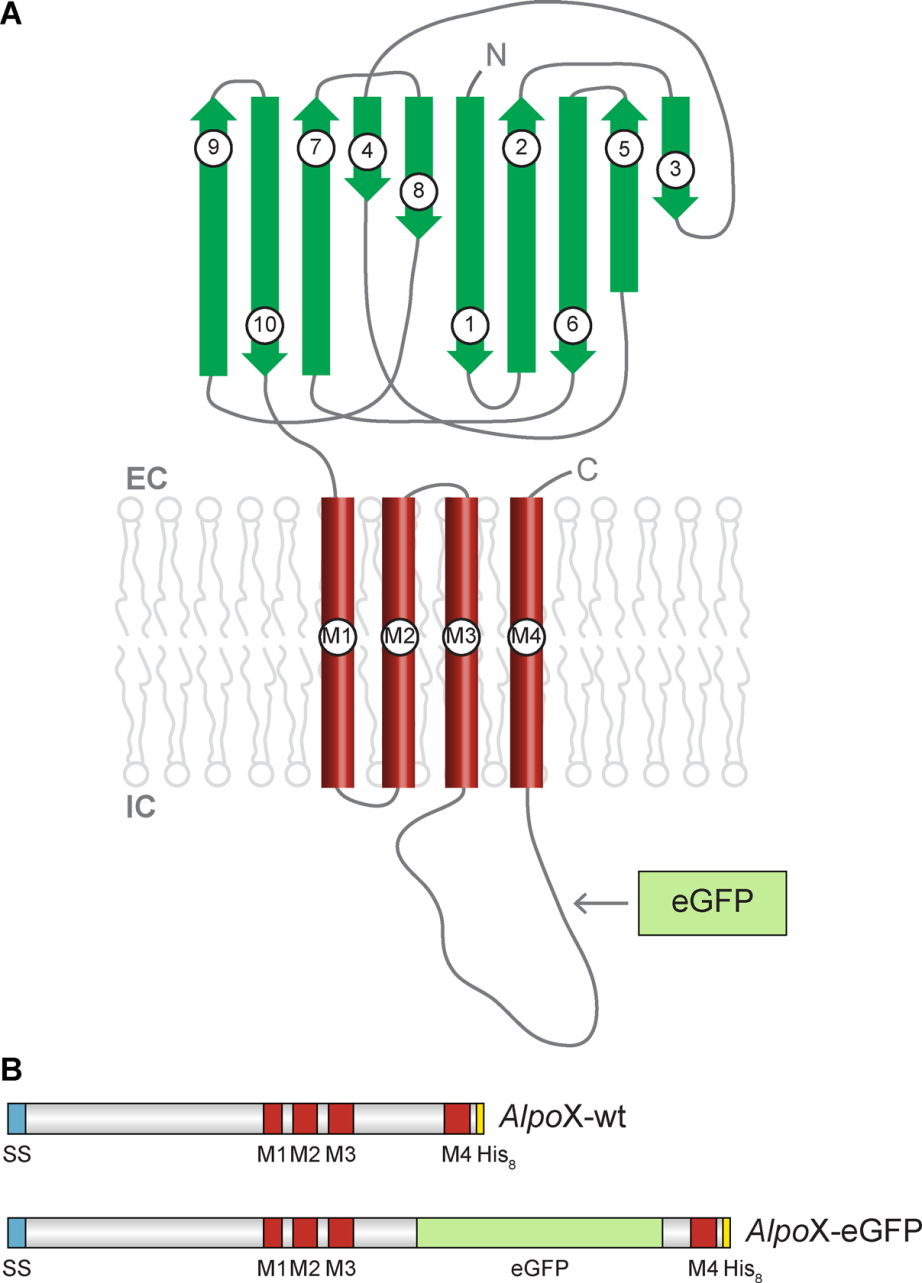
Subunit topology and construct design. β-strands are indicated in dark green, α-helices in red and eGFP in mint green. The signal sequence is colored blue, the His_8_-tag yellow. EC: extracellular, IC: intracellular. (A) Cartoon representation of a single pLGIC subunit, seen parallel to the membrane plane. The arrow indicates the position of eGFP in the *Alpo*X-eGFP constructs. (B) Construct design of *Alpo*X-wt and *Alpo*X-eGFP.

All these studies have indicated that the overall architecture is conserved among different members of the pLGIC family (Fig 1A) [20]. Each pLGIC is composed of five subunits symmetrically arranged around a central pore, forming the ion-conducting pathway. In its turn, each subunit consists of three distinct domains: an extracellular domain (ECD), a transmembrane domain (TMD) and an intracellular domain (ICD). The tertiary structure of the ECD is dominated by an arrangement of β-strands connected via loops. These loops, together with β-strands from the adjacent subunit form the ligand-binding pocket. The TMD contains four α-helices (M1-4). The M2 helix lines the ion channel pore. It contains the channel gate and is important for ion selectivity. The ICD, a loop connecting transmembrane helices M3 and M4, is uniquely found in eukaryotic CLRs where it influences ion conductance, receptor trafficking and assembly [20]. Unfortunately, the structural insights about this domain are rather limited today.

Based on the available structural information and on supplementary functional data, general hypotheses have been generated concerning ligand recognition, channel gating and ion conductance. However, it remains unclear whether these hypotheses are applicable to other members of the family with unknown structures and limited sequence identity. Additional structures, preferably in different conformational states and including the ICD, will validate these hypotheses and might ultimately lead to more adequate models to perform structure-based drug design.

Therefore we turned our attention towards homologues derived from the extremophilic organism *Alvinella pompejana*. *Alvinella pompejana* is a marine, polychaete worm that thrives 2000–3000 meter beneath sea level in hydrothermal vents with high sulfur and heavy metals concentration and is one of the most heat tolerant eukaryotes known to date [21,22]. It has been shown that proteins from extremophilic organisms display superior stability under laboratory conditions, making them ideal candidates for structural studies [23–26]. The potential application of *Alvinella pompejana* CLRs in structural studies was previously recognized by Juneja, P. *et al* [27]. They identified two CLR homologues: Alv-a9 and Alv-a1-pHCl. According to our nomenclature, Alv-a9 corresponds to *Alpo*1 and Alv-a1-pHCl corresponds to *Alpo*7. For Alv-a9 no activating ligand was found, whereas Alv-a1-pHCl is pH-activated [27], similar to ion channels found in other invertebrates [28]. In our study, we extended on these observations and identified five additional *Alvinella pompejana* CLR homologues. Our goal thus became to characterize these homologues both biochemically and functionally in the framework of future structural studies.

## Materials and Methods

### Bio-informatics

The protein database of *Alvinella pompejana* (http://jekely-lab.tuebingen.mpg.de/) was screened for CLR homologues by application of the Basic Local Alignment Search Tool algorithm (BLASTp) [29] with several human CLR sequences as search models. To analyze the primary structure of the identified homologues, a multiple sequence alignment was calculated with ClustalO [30] and Jalview [31]. In addition to mature sequences of these homologues, this alignment includes mature sequences of eukaryotic CLRs with known structure and of human CLR subunits displaying high sequence identity with the identified homologues. Simultaneously, the secondary structure was predicted with Phobius [32] and TMPred [33] and compared to the conserved general fold of known CLRs. Additionally, a pairwise sequence identity diagram was generated with ClustalO [30] and a cladogram was calculated (http://www.phylogeny.fr). Both this cladogram and the pairwise sequence identity diagram were generated based upon mature sequences.

### Construct design

The genetic sequences derived from the database of *Alvinella pompejana* were analyzed and optimized. Since TMpred, Phobius and the multiple sequence alignment indicated that *Alpo*3 only contained transmembrane helices M1-M3, this homologue was excluded from further experiments. In case of *Alpo*6 a native signal sequence was missing, therefore the signal sequence of the human glycine receptor subunit α1 (GLRA1) was added. To facilitate purification, a His_8_-tag was added to the C-terminus of each construct. The genes were synthesized by GenScript with optimized codon usage for expression in *Spodoptera frugiperda* 9 (*Sf*9) insect cells. These constructs were labeled “*Alpo*X-wt” with X referring to one of the homologues and varying between 1 and 7, excluding *Alpo*3 (Fig 1B).

For the purpose of a small-scale detergent screen by fluorescence size-exclusion chromatography (FSEC) [34] and an FSEC-based thermostability assay (FSEC-TS) [35], the genes of interest were fused to the enhanced Green Fluorescent Protein (eGFP). More precisely, eGFP was inserted in the intracellular loop between M3 and M4 in accordance with the strategy applied for GluCl and nAChR subunits [36,37]. These constructs were referred to as “*Alpo*X-eGFP” with X ranging between 1 and 7, excluding *Alpo*3 (Fig 1B). *Alpo*X indicates that experiments were both performed on “*Alpo*X-wt” and “*Alpo*X-eGFP”.

### Two-electrode voltage clamp

For the expression in *Xenopus laevis* oocytes, the genes coding for *Alpo*X-wt were subcloned into the vector pGEM-HE [38]. After *Nhe*I-treatment, the linearized plasmid DNA was transcribed to produce capped RNA by using the T7 mMESSAGE-mMACHINE transcription kit (Ambion). Four nanogram of the resulting RNA was injected into the cytosol of stage V and VI oocytes either manually or with an automated injection system (Roboinject, MultiChannelSystems). Oocyte preparations and injections were done using standard procedures [39]. Briefly, oocytes were harvested from ovarian lobes of female *Xenopus laevis* frogs, deeply anesthetized with MS-222 or tricaine. All these experiments conformed to the Geneva canton rules on animal experimentation (accreditation number G171/3551) or were approved by the KU Leuven Animal Facility (accreditation number P021/2013). Injected oocytes were incubated in a ND96-solution containing 96 mM NaCl, 2 mM KCl, 1.8 mM CaCl_2_, 2 mM MgCl_2_ and 5 mM HEPES, pH 7.4, supplemented with 50 mg/L gentamicin sulfate.

One to five days after injection, electrophysiological recordings were performed by conventional or automated TEVC (HiClamp, MultiChannel Systems). Cells were superfused with standard OR2 solution containing 82.5 mM NaCl, 2.5 mM KCl, 1.8 mM CaCl_2_, 1 mM MgCl_2_ and 5 mM HEPES buffered at pH 7.4. Unless indicated otherwise, cells were held at a fixed potential of –80 mV throughout the experiment. To minimize heat shock, all preparation and recording procedures were carried out at 16°C [40]. Functional characterization consisted of the screening of potential ligands, the establishment of concentration-activation curves and the determination of ion selectivity. Different small molecules were tested for their effects on *Alpo*X-wt, such as dopamine, taurine, γ-aminobutyric acid (GABA), glycine and ivermectin (the complete list and the corresponding concentrations are mentioned in Table 1). To determine concentration-activation curves, cells were exposed to increasing concentrations of the test substance. To assess ion selectivity, current-voltage (I-V) relationships were determined in control OR2 solution as well as in medium in which chloride ions were substituted by gluconate. These I-V curves were generated by voltage ramps applied within 4 s and constructed around the reversal potential (E_rev_ ± 80 mV). Leak properties of the cells measured in control conditions were subtracted from the I-V curves measured during exposure to 10 mM GABA.

**Table 1:** List of 33 chemical compounds and their concentration used for ligand screening by TEVC.

Test substance	Concentration (mM)
Acetylcholine chloride	10
Adenosine 5′-triphosphate (ATP) disodium salt hydrate	10
Amiloride hydrochloride hydrate	1
AMPA	1
Bradykinin acetate salt	1
Dopamine hydrochloride	10
GABA	10
Glycine	10
Histamine dihydrochloride	10
Imidacloprid	1
Ivermectin	1
Kainic acid monohydrate	1
L-Glutamic acid monosodium salt hydrate	10
Levamisol hydrochloride	10
Morantel citrate salt	1
Moxidectin	1
Muscarine chloride hydrate	10
Neurokinin A	1
Nicotine	1
Norepinephrine bitartrate salt	1
Octopamine hydrochloride	1
OR2 pH 5.0	
OR2 pH 8.0	
PNU-120596	1
Praziquantel	1
Pyrantel citrate salt	1
Serotonin creatinine sulfate	1
Strychnine	1
Substance P acetate salt hydrate	1
Taurine	1
Tryptamine hydrochloride	1
Tubocurarine	1
Tyramine	1

Data were acquired either on a manual setup or on an automated recording system (HiClamp, MultiChannel Systems). For the manual setup, data were offline filtered and subsequently processed with Clampfit 10.3 (Molecular Devices), Excel 2011 (Microsoft) and Prism 6.0 (GraphPad). Data acquired with the HiClamp were analyzed using the corresponding software. Concentration-activation curves were fitted with the empirical Hill equation where y is the normalized current amplitude, EC_50_ is the concentration for 50% activation and nH is the Hill coefficient.

$$y=\frac{1}{1+\frac{{EC}_{50}}{{\left[agonist\right]}^{nH}}}$$

### Expression and small-scale detergent screen

Expression of *Alpo*X-wt and *Alpo*X-eGFP in *Sf*9 insect cells was driven by the Bac-to-Bac® Baculovirus expression system (Invitrogen). The constructs were subcloned into the pFastBac-1 vector followed by 3 rounds of baculovirus amplification. Insect cells were infected at a density of 1–3 million cells per mL and harvested by centrifugation 60–72 hours after infection (10,000 g for 15 min). Harvested cells were resuspended in buffer A (150 mM NaCl, 50 mM Na phosphate, pH 7.4) supplemented with a cocktail of protease-inhibitors (1 mM PMSF, 1 μg/mL aprotinin, 1 μg/mL leupeptin and 1 μg/mL pepstatin), 20 μg/mL DNase and 5 mM MgCl_2_. These resuspended cells were lysed through high pressure homogenization (Emulsiflex C5, Avestin). Membranes were subsequently isolated by ultracentrifugation (125,000 g for 40 min) and resuspended in an equal volume of buffer A supplemented with protease inhibitors.

Membrane suspensions derived from *Alpo*X-eGFP constructs were used for a small-scale detergent screen by FSEC [34]. Membrane aliquots were solubilized for 90 min with a wide variety of detergents and detergent mixtures (Anatrace) at a final concentration amply exceeding the critical micelle concentration (CMC) (S1 Table). The detergents tested, were: n-dodecyl-β-D-maltopyranoside (DDM), n-undecyl-β-D-maltopyranoside (UDM), n-decyl-β-D-maltopyranoside (DM), n-octyl-β-D-glucopyranoside (OG), n-nonyl-β-D-glucopyranoside (NG), N,N-dimethyldodecylamine N-oxide (LDAO), 3[(3-cholamidopropyl) dimethylammonio]propanesulfonic acid (CHAPS), 3-[(3-cholamidopropyl)dimethylammonio]-2-hydroxy-1-propanesulfonate (CHAPSO), decyl maltose neopentyl glycol (DMNG), lauryl maltose neopentyl glycol (LMNG) and a mixture of LMNG and CHAPS. After ultracentrifugation (30,000 g for 40 min), the solubilized fractions were loaded on the FSEC system, consisting of a Superose 6 10/300 column (GE Healthcare) coupled to a fluorescence detector (Rf10Axl, Shimadzu). The running buffer consisted of buffer A, supplemented with the corresponding detergent at a concentration slightly exceeding the CMC (S1 Table).

### Large-scale purification

Crude membranes, obtained after high pressure homogenization and ultracentrifugation of *Sf*9 insect cells expressing *Alpo*X, were resuspended in buffer A, supplemented with protease-inhibitors and solubilized by the use of FSEC identified detergents (S1 Table) at 4°C during 90 min. The unsolubilized material was removed by ultracentrifugation at 30,000 g for 40 min. Subsequently, the supernatant was incubated with Ni Sepharose High Performance beads (GE Healthcare) at 4°C for 90 min whereafter the beads were washed with buffer A containing detergent, protease-inhibitors and increasing concentrations of imidazole. The protein of interest was eluted with buffer A containing detergent, protease-inhibitors and 300 mM imidazole. Finally, the eluted protein was applied on the size-exclusion chromatography (SEC) system, consisting of a Superose 6 10/300 column (GE Healthcare) coupled to a UV-detector (Monitor UV-900, GE Healthcare). The running buffer consisted of buffer B (150 mM NaCl, 10 mM Na phosphate, pH 7.4) supplemented with the corresponding detergent at a concentration slightly above the CMC (S1 Table). Eluted fractions were analyzed by SDS-PAGE.

To improve the monodispersity of *Alpo*6, additional purifications were performed in the presence of the identified ligands. These ligands were added at a concentration of approximately 5 x EC_50_ (1 mM taurine, 30 mM GABA or 100 mM glycine) during every step of the purification protocol.

### Negative stain EM

Samples for negative stain electron microscopy (EM) were prepared by applying 2 μL of protein solution (0.03 mg/mL) on glow discharged carbon-coated copper grids. After three short wash-steps with Milli-Q water, samples were stained with 1% uranyl formate solution and dried. The grids were imaged in an electron microscope (JEOL JEM-1400) equipped with a LaB_6_ cathode and operated at 120 kV. Images were recorded with a 4096 x 4096 pixel CMOS TemCam-F416 camera (TVIPS) at a nominal magnification of 50,000 and a corresponding pixel size of 2.29 Å under a defocus between 2.5 and 4.0 μm. For calculating 2D images, particles were selected from micrographs in e2boxer [41]. The phase flipped images were aligned and classified in SPARX [42].

### FSEC-based thermostability assay

An FSEC-based thermostability assay (FSEC-TS) was performed [35] to determine the thermal stability of the purified *Alpo* CLR homologues. Aliquots of purified *Alpo*1-eGFP in DDM, *Alpo*4-eGFP in LMNG-CHAPS and *Alpo*6-eGFP in LMNG were heated at different temperatures in the range of 4–95°C for 10 min, centrifuged at 87,000 g for 20 min and applied on the FSEC system. The relative oligomeric peak heights, normalized with respect to the peak height derived from the 4°C sample, were plotted against the corresponding temperatures. Melting curves were fitted through the data points with a sigmoidal curve (Prism6, GraphPhad) and the corresponding melting temperatures (T_m_) were determined.

Similarly, FSEC-TS was applied to quantify the effect of ligands on the thermal stability of purified *Alpo*6-eGFP. Aliquots of *Alpo*6-eGFP purified both in the presence and absence of ligands were incubated at 4°C or 65°C during 10 min. The resulting samples were centrifuged (87,000 g for 20 min) and analyzed by FSEC. The oligomeric peak heights were normalized against the signals derived from the corresponding samples incubated at 4°C. The data were plotted as a histogram, which allowed a quantitative comparison of the thermal stability of *Alpo*6-eGFP in the presence and absence of different ligands.

### Nanobody production, purification and screening

Nanobodies directed against *Alpo*1 were produced by immunization of llamas (http://steyaertlab.structuralbiology.be/). Briefly, one llama (*Lama glama*) was immunized six times with 900 μg of DDM-solubilized *Alpo*1-wt in total. Lymphocytes of the anticoagulated blood of the immunized llama were used to prepare cDNA, which served as a template to amplify the open reading frames coding for the variable domains of the heavy-chain antibodies, also called Nanobodies. The Nanobody repertoire was cloned into phage-display vector pMESy4 [43]. After one round of biopanning on either solid-phase-coated *Alpo*1-wt or antibody trapped *Alpo*1-eGFP a clear enrichment of *Alpo*1 specific phage was observed. 184 randomly chosen colonies were grown for expression of their Nanobody as soluble protein. Crude periplasmic extracts were tested by ELISA and 115 extracts were shown to be specific toward *Alpo*1. From the positive clones, the Nanobody open reading frames were amplified by PCR and their sequence was analyzed. Thirty-nine distinct families were revealed.

Heterologous expression and purification of these Nanobodies were performed as previously described [43]. To identify Nanobodies that are suitable as crystallization chaperones and bind with a high stoichiometric ratio (number of Nanobodies per *Alpo*1 oligomer), a small-scale FSEC-based screening was carried out. The purified Nanobodies were added to purified *Alpo*1-eGFP and incubated at room temperature during 1 hour followed by FSEC analysis of the resulting complexes. Additionally, FSEC-TS experiments were performed to assess the possible stabilizing effects of the identified Nanobodies. Therefore *Alpo*1-eGFP-Nb complexes were heated at 65°C for 10 min, centrifuged (87,000 g for 20 min) and analyzed by FSEC. The relative peak heights of the heated complexes were compared to the relative peak heights of control samples, which were heated in the absence of Nanobody.

## Results

### Seven CLR homologues are identified in the proteome of *Alvinella pompejana*

**Fig 2:**
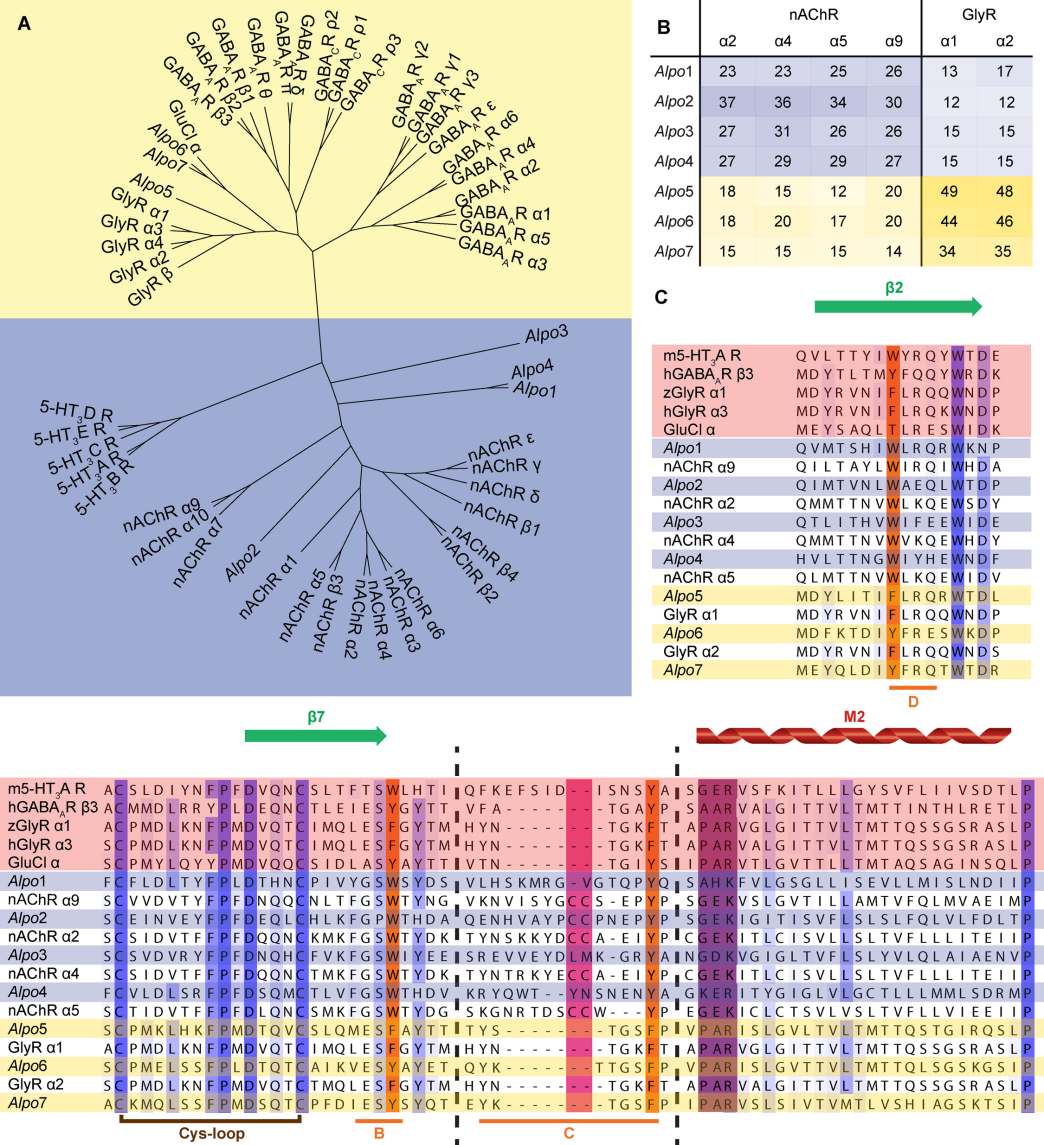
Sequence analysis of *Alpo* CLR homologues. Anion-selective channels are indicated in yellow, (putative) cation-selective channels in blue. (A) Cladogram displaying the relationship between *Alpo*, known human CLR subunits and GluCl. (B) Pairwise sequence identity diagram of *Alpo* and the most closely related known human CLR subunits. The degree of sequence identity is displayed in shades of blue for putative cation-selective channels and in shades of yellow for anion-selective channels. (C) Multiple sequence alignment including sequences of *Alpo* (blue and yellow), CLRs with known structures (pink) and human CLR subunits with high sequence identity to the identified homologues. The degree of amino acid conservation is displayed in shades of blue, β-strands are indicated in green and the M2 helix in red. Secondary structure elements are retrieved from the m5-HT_3_A R crystal structure [17]. Conserved aromatic residues and loops B, C and D involved in ligand binding are colored in orange, the vicinal disulphide is indicated in fuchsia, the ion selectivity filter is shown in purple and the eponymous Cys-loop in brown.

The BLASTp search in the proteome of *Alvinella pompejana* resulted in the identification of seven CLR homologues, which we named *Alpo*1-7. The protein sequences have been deposited in the protein database of The Max Planck Institute for Developmental Biology (http://jekely-lab.tuebingen.mpg.de/) under accession numbers P74014 (*Alpo*1), P64117 (*Alpo*2), P61179 (*Alpo*3), P60768 (*Alpo*4), P65805 (*Alpo*5), P52796 (*Alpo*6) and P60838 (*Alpo*7). *Alpo*1 is equal to Alv-a9 and *Alpo*7 is equal to Alv-a1-pHCl as reported by Juneja, P. *et al* [27]. The cladogram provides a visual representation of the relationship between *Alpo* CLR homologues, known human CLR subunits and GluCl (Fig 2A). Mature sequences of *Alpo*1-4 were clustered together with cation-selective members of the CLR family, whereas *Alpo*5-7 appeared to be more closely related to anion-selective members. Furthermore, a pairwise sequence identity diagram was calculated to quantify the overall amino acid conservation between *Alpo* CLR subunits and human CLR subunits (Fig 2B). On average, mature sequences of *Alpo*1-4 share 28% sequence identity with human nAChR subunits α2, α4, α5 or α9, whereas the average sequence identity of *Alpo*5-7 with human GlyR subunits α1 or α2 is 43%. The amino acid conservation is outlined in more detail in the multiple sequence alignment, where some of the key regions involved in channel function, are highlighted (Fig 2C, S1 Fig). Additionally, at least one of the secondary structure prediction tools confirmed the presence of four transmembrane helices for all *Alpo* CLR subunits except for *Alpo*2 and *Alpo*3. For *Alpo*3 three transmembrane helices were predicted whereas five transmembrane helices were predicted for *Alpo*2.

The predicted length of the ICD between M3 and M4 varies from 11 up to 118 amino acids.

### *Alpo*5-wt and *Alpo6*-wt can be activated by glycine, GABA and taurine

**Fig 3:**
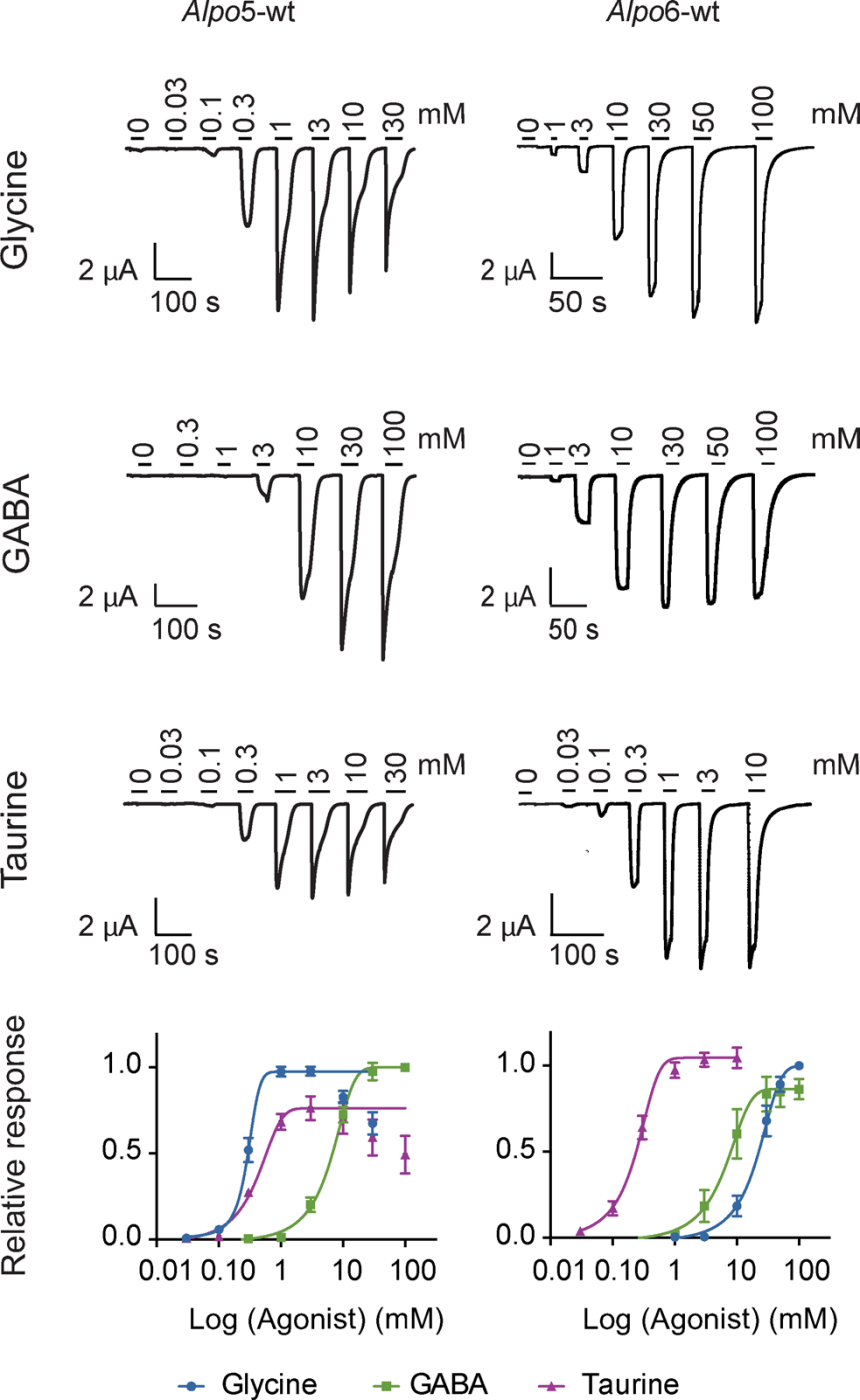
Functional characterization of *Alpo*5-wt and *Alpo*6-wt by TEVC. Top: Currents derived from the activation of *Alpo*5-wt and *Alpo*6-wt by increasing concentrations of glycine, GABA or taurine. Bottom: Concentration-activation curves of glycine (blue), GABA (green) and taurine (purple) for *Alpo*5-wt and *Alpo*6-wt.

The effects of pH and different small molecules, including neurotransmitters and allosteric modulators, on *Alpo*X-wt were tested with TEVC (Table 1). *Alpo*5-wt and *Alpo*6-wt could both be activated by the neurotransmitters glycine, GABA and taurine (Fig 3). Upon determination of concentration-activation curves of *Alpo*5-wt, we found that both glycine and GABA were full-agonists of the channel. A plot of the peak inward current as a function of the logarithm of the GABA concentration yielded a typical concentration-activation curve that was readily fitted with a single Hill equation with an EC_50_ value of 6.980 ± 0.512 mM and a Hill coefficient of 2.49 ± 0.09 (n = 4). Similarly, the concentration-activation relationship for glycine was characterized by an EC_50_ value of 291.0 ± 16.4 μM and a Hill coefficient of 2.68 ± 0.08 (n = 4). Taurine, on the other hand, was a partial agonist of *Alpo*5-wt. On average, the currents evoked by taurine at a saturating concentration were 74% of the responses evoked by a saturating concentration of glycine. The concentration-activation relationship for taurine was characterized by a single Hill equation with an EC_50_ value of 370.0 ± 30.2 μM and a Hill coefficient of 2.51 ± 0.18 (n = 4). In case of *Alpo*6-wt, glycine and taurine acted as full agonists, whereas GABA exhibited a partial agonistic effect. On average, the current evoked by a saturating concentration of GABA corresponded to 86% of the current evoked by a saturating concentration of glycine. The channel was most sensitive to taurine, reflected by an EC_50_ value of 226.7 ± 30.5 μM (n = 3). The affinities of *Alpo*6-wt for glycine and GABA were reflected by EC_50_ values of 22.14 ± 4.53 mM (n = 3) and 6.965 ± 1.590 mM (n = 3), respectively. The Hill-coefficients of the corresponding activation-response curves were 1.87 ± 0.12 for taurine, 2.20 ± 0.16 for glycine and 1.94 ± 0.15 for GABA.

**Fig 4:**
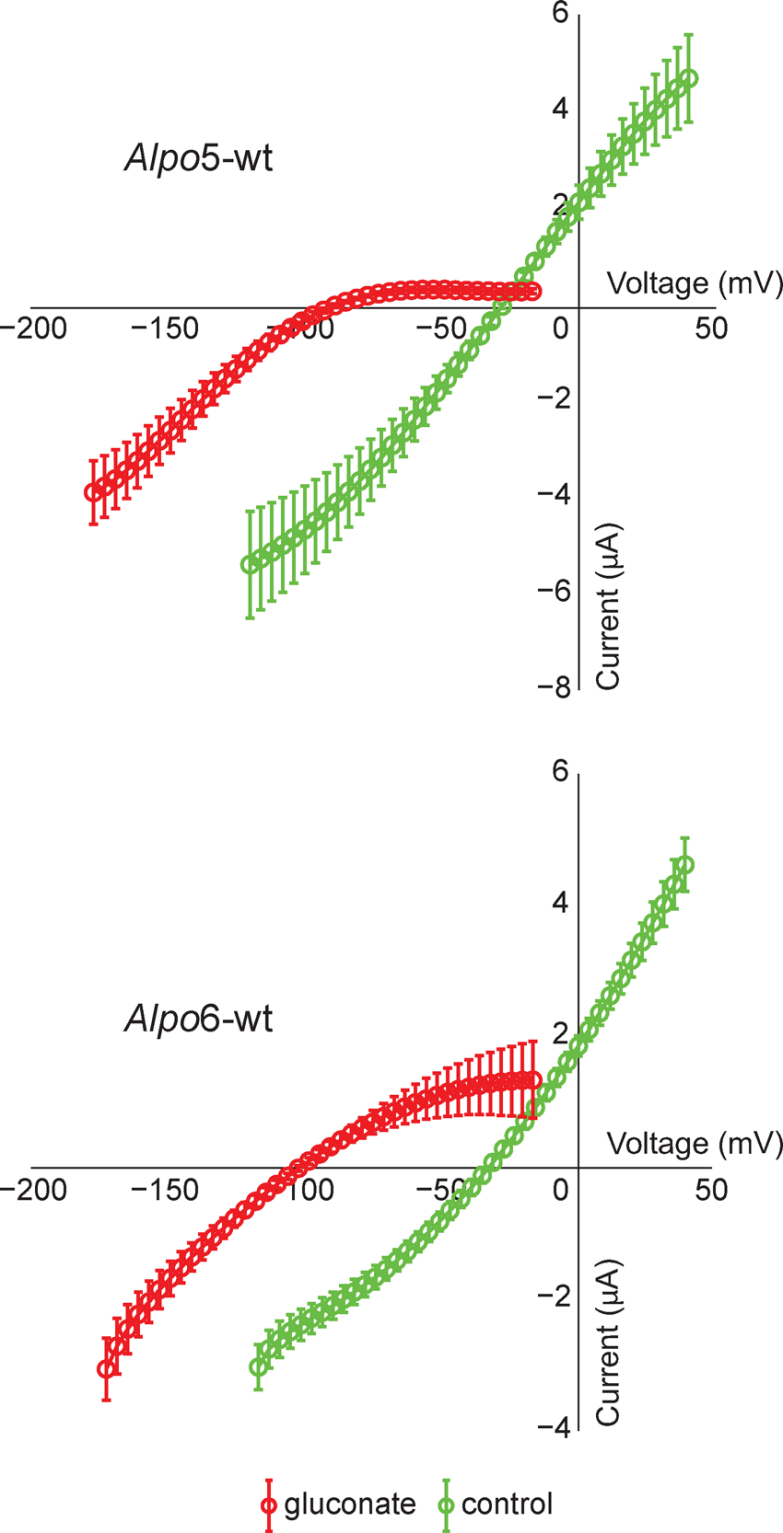
Ion selectivity of *Alpo*5-wt and *Alpo*6-wt. Current-voltage curves of *Alpo*5-wt and *Alpo*6-wt activated by 10 mM GABA recorded in the presence of extracellular chloride (green) or gluconate ions (red).

Furthermore, we determined the ion selectivity of *Alpo*5-wt and *Alpo*6-wt (Fig 4). Results obtained in five cells were averaged for *Alpo*5-wt and showed that substitution of the chloride ions by the non-permeable anion gluconate in the extracellular medium caused a leftward shift of the reversal potential indicating that the channel was permeable to anions. Results obtained for *Alpo*6-wt yielded the same conclusions. In addition to a leftward shift of the I-V curves, an apparent change in rectification occurred from linear in control conditions to inward rectifying in the presence of gluconate. This can be explained by a decrease of conductance at potentials above E_rev_ due to the absence of permeable anions in the extracellular medium.

Moreover, we could confirm the activation of *Alpo*7-wt by elevated proton concentrations as previously described by Juneja, P. *et al* [27]. For *Alpo*1-wt, *Alpo*2-wt and *Alpo*4-wt none of the tested substances (Table 1) evoked channel activation.

### Several *Alvinella pompejana* CLR homologues can be purified in a monodisperse, oligomeric state

**Fig 5:**
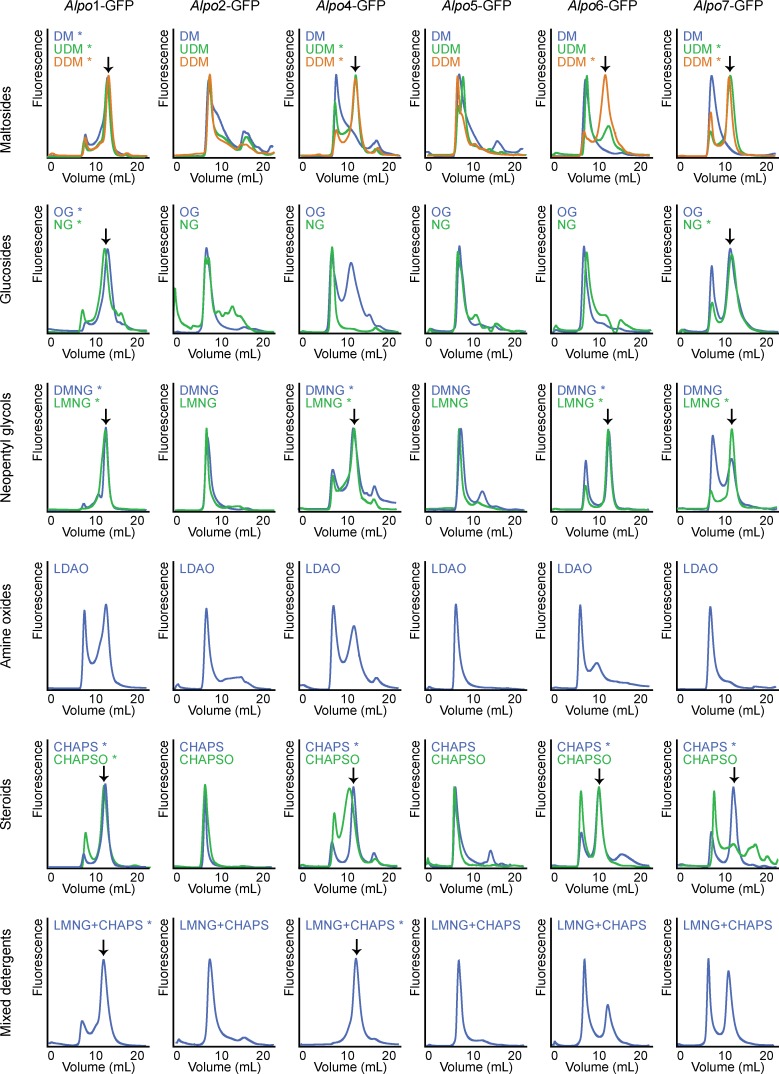
FSEC-based detergent screen [34]. FSEC profiles of *Alpo*X-eGFP solubilized by the indicated detergents. The fluorescent signals were approximately equated with each other. * indicates a suitable detergent for the solubilization of the particular protein. A detergent was considered suitable if FSEC resulted in a chromatogram characterized by a high, symmetric peak around 13.5 mL and a lower peak around the void volume (7 mL).

Upon expression of the *Alpo* CLR homologues in *Sf*9 insect cells, an FSEC-based detergent screen was performed to identify detergents that extract these membrane proteins from the lipid bilayer in a monodisperse and oligomeric state (Fig 5). Several detergents from different classes were tested including maltosides, glucosides, neopentyl glycols, steroid-derivatives and mixed detergents. A detergent was considered suitable for a particular protein if FSEC resulted in a chromatogram characterized by a high, symmetric peak around 13.5 mL and a lower peak around the void volume (7 mL). According to calibration of the Superose6 10/300 column, a peak around 13.5 mL corresponds to detergent-solubilized, oligomeric protein, whereas a peak around 7 mL accounts for protein aggregates.

Detergent-solubilization of *Alpo*2-eGFP and *Alpo*5-eGFP mainly resulted in the absence of a symmetric, oligomeric peak and a high amount of protein aggregates, rendering it impossible to perform large-scale purifications of these homologues. By contrast, multiple suitable detergents were identified for the four remaining homologues: *Alpo*1-eGFP, *Alpo*4-eGFP, *Alpo*6-eGFP and *Alpo*7-eGFP, as was indicated by FSEC profiles displaying a limited amount of aggregates, eluting in the void volume, and a high, symmetric peak around 13.5 mL (Fig 5).

**Fig 6:**
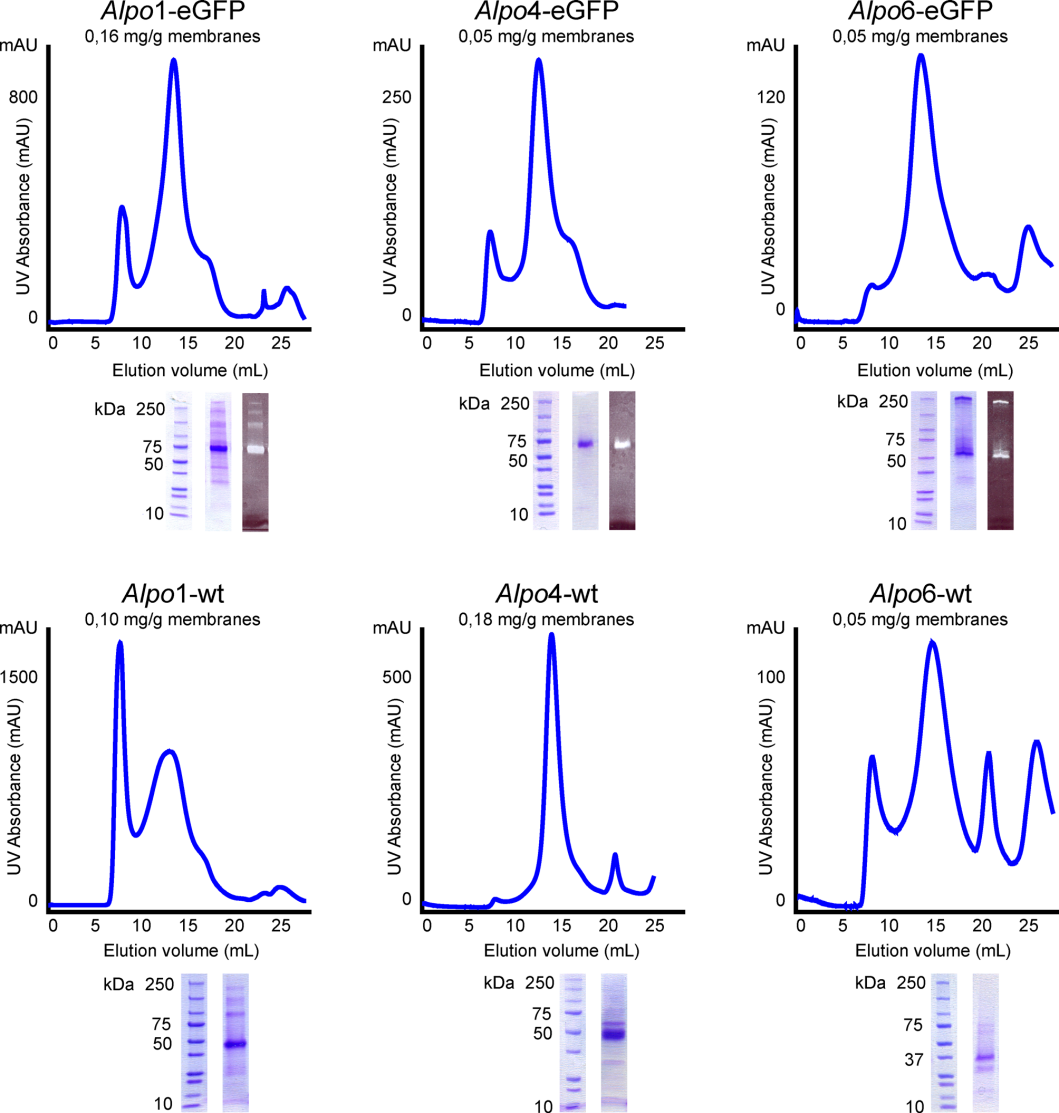
Large-scale purification of *Alpo*1, *Alpo*4 and *Alpo*6. SEC-profiles of purified *Alpo* CLR homologues and SDS-PAGE analyses of the corresponding oligomeric peak fractions. Left: Purification of Alpo1-eGFP and *Alpo*1-wt in DDM. Middle: Purification of *Alpo*4-eGFP and *Alpo*4-wt in LMNG-CHAPS. Right: Purification of *Alpo*6-eGFP and *Alpo*6-wt in the presence of glycine and LMNG.

Once suitable solubilization conditions were identified, large-scale purifications were performed for *Alpo*1, *Alpo*4 and *Alpo*6. For *Alpo*7 no purification efforts were undertaken given the recent publication of preliminary biochemical data describing the expression and purification of this protein [27]. The overall purification strategy was similar for all three constructs and consisted of a 2-step purification protocol composed of immobilized metal affinity chromatography (IMAC) and SEC. We obtained sufficient amounts of oligomeric protein for *Alpo*1-wt, *Alpo*1-eGFP, *Alpo*4-wt and *Alpo*4-eGFP with typical yields varying between 0.05 mg and 0.18 mg of SEC-purified protein/g membranes (Fig 6). SEC of the GFP-tagged constructs, *Alpo*1-eGFP and *Alpo*4-eGFP, resulted in monodisperse, oligomeric protein indicated by a main peak with a retention volume of approximately 13.5 mL (Fig 6). Notwithstanding that a minor shoulder around 17.5 mL was observed, the main fraction of the peak did appear to contain monodisperse protein. By contrast, important differences in monodispersity were observed for the corresponding wild type constructs (Fig 6). Purification of *Alpo*4-wt resulted in a highly symmetric, oligomeric peak during SEC, whereas the SEC profile of *Alpo*1-wt displayed a broader, oligomeric peak with a pronounced shoulder on the left, indicative for the co-elution of several oligomeric states.

Large-scale purification of *Alpo*6-eGFP typically resulted in a low amount (0.03 mg of SEC-purified protein/g membranes) of polydisperse protein, not amenable for structural studies (S2A Fig). To improve the biochemical behavior of *Alpo*6, additional purifications were performed in the presence of the identified ligands (S2 Fig). The addition of taurine and GABA did not improve protein yields nor homogeneity (S2C and S2D Fig), whereas the addition of glycine resulted in a more symmetric oligomeric peak both for *Alpo*6-eGFP as well as for *Alpo*6-wt (S2B and S2E Fig). The protein yields remained low, 0.05 mg/g membranes, but are sufficient for future structural studies. The beneficial effect of glycine on *Alpo*6 was further substantiated by FSEC-TS (S2F Fig). Heating *Alpo*6-eGFP to 65°C during 10 min, resulted in a 44 ± 3% decrease in fluorescent signal measured as oligomeric peak height. In contrast, no loss of fluorescent signal was observed for heated *Alpo*6-eGFP purified in the presence of glycine. We could therefore conclude that glycine had a significant effect on the thermal stability of *Alpo*6.

To determine the biochemical purity of the SEC-purified proteins, the peak fractions were subjected to SDS-PAGE analysis (Fig 6). A pronounced band was observed around the expected molecular weight mark of an *Alpo* monomer, more precisely around the 50 kDa mark for *Alpo*1-wt (48.5 kDa) and *Alpo*4-wt (50.9 kDa) and around the 75 kDa mark for *Alpo*1-eGFP (75.4 kDa) and *Alpo*4-eGFP (77.8 kDa). For *Alpo*6-eGFP (70.7 kDa) and *Alpo*6-wt (43.8 kDa) the monomeric band appeared lower than expected, around the 50 kDa mark and around the 37 kDa mark respectively. This gel shift is not uncommon for SDS-PAGE analysis of membrane proteins. A faster migration can be due to an incomplete unfolding of the protein in SDS-buffer or possibly due to an altered number of bound SDS-molecules relative to the size of the protein [44]. In case of *Alpo*X-eGFP, the pronounced monomeric band lighted up fluorescent upon excitation. For *Alpo*1-wt, *Alpo*1-eGFP and *Alpo*6-eGFP additional bands could be observed between the 150 kDa and 250 kDa mark. Since these bands lighted up fluorescent in case of the GFP-tagged constructs, we can ascribe these bands to oligomeric assemblies or protein aggregates (Fig 6).

**Fig 7:**
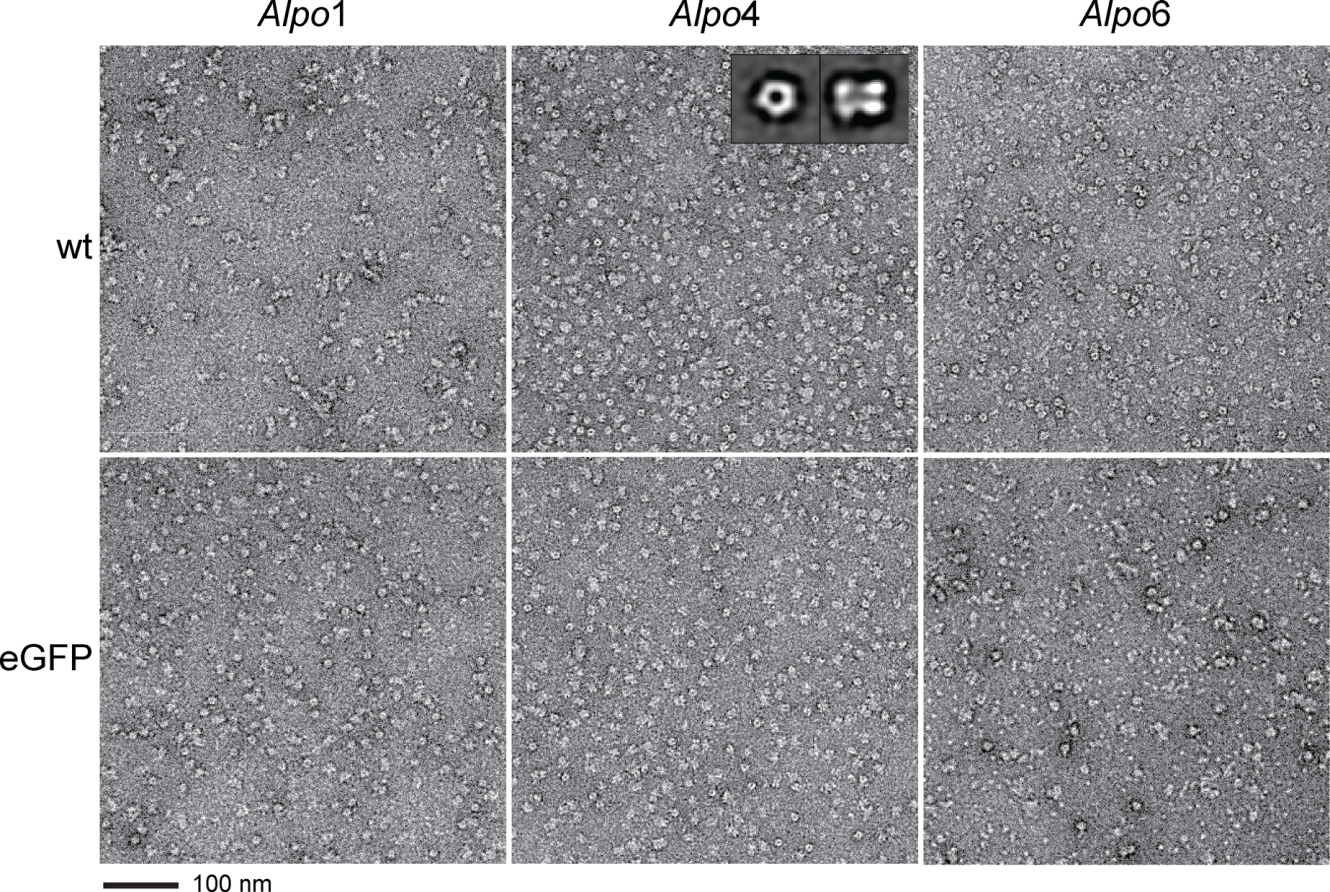
Negative stain electron microscopy. Representative areas of micrographs with negatively stained purified *Alpo*1-wt, *Alpo*1-eGFP, *Alpo*4-wt, *Alpo*4-eGFP, *Alpo*6-wt and *Alpo*6-eGFP. The insets in *Alpo*4-wt show class averages corresponding to top and side views of the protein. The edge length of the insets is 220 Å.

Furthermore, characterization of purified *Alpo*1, *Alpo*4 and *Alpo*6 was conducted using negative stain transmission EM (Fig 7). The micrographs mainly visualized single monodisperse protein particles in two preferential orientations consistent with side and top views. Two-dimensional class averages calculated for the best behaving construct, *Alpo*4-wt, displayed characteristic top views indicating five-fold rotational symmetry with a diameter of 90 ± 10 Å and characteristic side views in the shape of elongated tube-like structures of 120 ± 10 Å long consisting of two fragments, putatively corresponding to the TMD and ECD, with a channel running through the center of the tube. The dimensions of the channel are in agreement with the dimensions of the glycine α1 receptor with a diameter around 75 Å and a height of 110 Å [18].

Overall, the negative stain EM-images are consistent with the SEC-results. Micrographs of *Alpo*1 for example, indicate that *Alpo*1-wt is less monodisperse than *Alpo*1-eGFP by forming higher oligomers through interaction of hydrophilic regions. These interactions are disrupted by insertion of eGFP. Additionally, the improved purification protocol of *Alpo*6-wt by the addition of glycine, clearly resulted in monodisperse protein as shown by negative stain EM. However, this was not the case for *Alpo*6-eGFP with micrographs displaying aggregates and smaller particles, which may correspond to residual impurities or degraded protein.

### Purified *Alvinella pompejana* CLR homologues are thermostable

**Fig 8:**
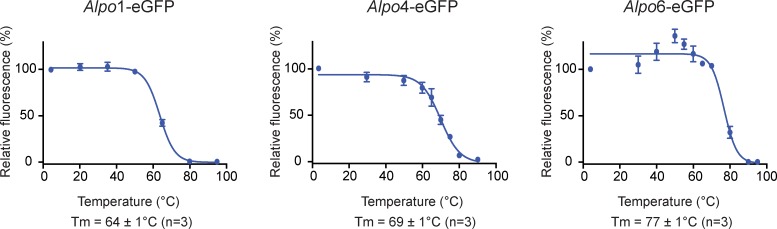
Melting curves of purified *Alpo*1-eGFP, *Alpo*4-eGFP and *Alpo*6-eGFP. Melting curves of *Alpo*1-eGFP, *Alpo*4-eGFP and *Alpo*6-eGFP purified in DDM, LMNG-CHAPS and LMNG-glycine respectively, generated by FSEC-TS [35]. The deduced melting temperatures are indicated (n = 3).

By implementation of FSEC-TS, we determined melting curves of purified *Alpo*1-eGFP in DDM, *Alpo*4-eGFP in LMNG-CHAPS and *Alpo*6-eGFP in the presence of glycine and LMNG. The corresponding melting temperatures were 64 ± 1°C, 69 ± 1°C, and 77 ± 1°C (n = 3), respectively (Fig 8). We hypothesized that the thermal stability of *Alpo*6-eGFP in presence of glycine might be hampered by the thermal stability of eGFP since its melting temperature is close to the reported melting temperature (76°C) of eGFP [35].

### Nanobody 9 stabilizes *Alpo1*-eGFP

In analogy with other eukaryotic CLRs for which the structure was solved [14,15,17], we implemented the use of crystallization chaperones and produced Nanobodies directed against *Alpo*1 [45–47]. Forty-one Nanobodies (Nanobody 1-Nanobody 41) with confirmed affinity for *Alpo*1 were discovered. To rationalize future co-crystallization trials, we performed an FSEC-based screen to determine which Nanobodies shift the retention volume of the oligomeric peak the most and thus probably bind with the highest stoichiometric ratio. Overall, the shift in retention volume was limited because of the low molecular weight of Nanobodies. However, for one Nanobody, Nanobody 9 (Nb9), the shift was more pronounced (0.7 mL) (S3A Fig). We further investigated whether this specific Nanobody would additionally thermostabilize the receptor. Therefore we applied three samples on FSEC-TS: one containing *Alpo*1-eGFP incubated at 4°C, one containing *Alpo*1-eGFP incubated at 65°C and one containing the *Alpo*1-eGFP-Nb9 complex incubated at 65°C. In this particular experiment the fluorescent signal of *Alpo*1-eGFP heated at 65°C was 56 ± 4% (n = 3) of the fluorescent signal of the 4°C sample. However, when we added Nb9 prior to heating, this fluorescent signal only decreased to 83 ± 5% (n = 3) (S3B Fig). This result indicated that Nb9 had a thermostabilizing effect on *Alpo*1-eGFP.

## Discussion

In our quest for CLR homologues suitable for structural studies, we characterized seven candidates, named *Alpo*1-7 derived from the extremophilic annelid, *Alvinella pompejana*. *Alvinella pompejana* also called the Pompeii worm, is one of the most heat-tolerant eukaryotes known to date [21,22]. It colonizes hydrothermal vents in the East Pacific Rise and thus thrives in hostile environments characterized by high temperatures and pressures, hypoxia and high concentrations of toxic compounds, like H_2_S. In 2010, a genome project indicated that this annelid has only known a slow rate of evolution [48]. More precisely, several genes absent in other Protostome organisms, but present in Deuterostomes, among which humans, appeared to be conserved in *Alvinella pompejana*. Subsequently, the authors hypothesized that thermostable proteins derived from this organism are ideal models to study human proteins. Given this high degree of conservation and their presumed thermostability, CLR homologues identified in the proteome of *Alvinella pompejana* appeared to be ideal candidates for future structural studies.

First clues regarding the functionality and ion selectivity of these homologues were derived from the cladogram and the pairwise sequence identity diagram dividing the *Alpo* CLR homologues over the cationic (*Alpo*1 to *Alpo*4) and anionic branch (*Alpo*5 to *Alpo*7) of the family. Remarkably, *Alpo*5 and *Alpo*6, share over 45% of sequence identity with the glycine receptor subunits α1 and α2. This exceeds the sequence identity of GluClα, derived from the Protostome *Caenorhabditis elegans*, with these receptors. Detailed information regarding amino acid conservation can be retrieved from the multiple sequence alignment. In the ECD, several aromatic residues, which were previously identified as molecular determinants for neurotransmitter binding, appear to be conserved among *Alpo*1-*Alpo*7. Furthermore, the eponymous disulfide bridge in between the sixth and the seventh β-strand is present as well. For *Alpo*2, we can even distinguish a vicinal disulfide in loop C, which is a prototypical feature in the neurotransmitter binding site of nAChR α subunits. Surprisingly, TMPred and Phobius predicted five transmembrane helices per monomer of *Alpo*2. A possible explanation for this aberrant feature can be that one of the predicted transmembrane helices is confused with the intracellular MX-helix. This hypothesis is supported by a lower score in the prediction output for one of the predicted transmembrane helices in comparison to the other helices. In addition, this particular helix aligns with the MX-helix of the mouse 5-HT_3_A receptor upon sequence alignment [17].

If we take a closer look at the ion selectivity filter, the above-mentioned hypothesis concerning ion selectivity can be further substantiated. Indeed, a formal negative charge in the -1’ position, typical for cation-selectivity, is present for all presumed cation-selective members, except for *Alpo*1. In case of the presumed anion-selective members, we can identify the signature sequence -PAR- in the M2 region of the pore domain, which is in agreement with previous findings [14,16]. To substantiate these hypotheses concerning ion selectivity and to functionally characterize these novel CLR homologues, electrophysiological recordings were performed. As expected by the exceptionally high sequence identity, *Alpo*5-wt and *Alpo*6-wt can both be activated by glycine, GABA and taurine and are both anion-selective. *Alpo*6 has mM affinity for GABA and glycine, however the affinity for taurine is in the μM range. The pharmacological profile of *Alpo*5 is strikingly similar to the profile of the human homopentameric glycine α1 receptor. Not only are both EC_50_ values for glycine in the μM range, the difference in affinity for glycine, GABA and taurine is also comparable (EC_50_ glycine < EC_50_ taurine < EC_50_ GABA) [49]. Upon till now, only one invertebrate, glycine-gated CLR homologue was identified, named Ci-GlyR. Ci-GlyR was found in the genome of *Ciona intestinalis*, a marine, Deuterostome invertebrate, and was thought to be involved in the organization of swimming [50]. However, *Alpo*5 and *Alpo*6 are the first glycine-gated CLR homologues derived from a non-Deuterostome organism. Therefore, we can hypothesize that the glycine receptor has an earlier evolutionary origin than assumed so far if we exclude the option of horizontal gene transfer [50].

Notwithstanding the recent publication of electron cryo-microscopic structures of the zebrafish glycine α1 receptor [18] and of an X-ray crystallographic structure of the human glycine α3 receptor [19], questions concerning the glycine receptor remain. As for example, how the ICD is involved in ion permeation and how glycine is recognized at the neurotransmitter binding site. A high-resolution three-dimensional structure of *Alpo*5 or *Alpo*6 in complex with glycine and/or showing the ICD can thus still be of high relevance in order to understand the molecular mechanism underlying ion channel function. For *Alpo*1-wt, *Alpo*2-wt and *Alpo*4-wt no activating compounds could be identified so far, and therefore we were unable to confirm their function as CLRs. Possibly, these homologues are activated by a yet to be identified agonist or are not properly expressed in *Xenopus* oocytes. Alternatively, they could be part of functional heteropentameric assemblies. In vertebrates for example, only subunits α7, α8 and α9 of the nAChR are able to form functional homopentamers [51,52]. Further investigation remains necessary. However, given the high percentages of sequence identity with human nAChR α subunits, high-resolution structures of *Alpo*1, *Alpo*2 or *Alpo*4, might extend current structural insights into nAChRs.

Besides functional assays, biochemical experiments were performed to assess the suitability of the identified homologues for structural studies. Therefore, we expressed these homologues in *Sf*9 insect cells, performed a detergent screen and carried out large-scale purifications. We firstly expressed the GFP-tagged constructs since this allowed us to conduct a small-scale FSEC-based detergent screen on non-purified membrane extracts [34]. The choice of a suitable detergent is crucial as the decrease of protein stability upon extraction from the lipid bilayer is a major bottleneck during membrane protein purification [53]. Several detergents from different classes including maltosides, glucosides, neopentyl glycols, amine oxides, bile acid derivates and mixed detergents were tested. Strikingly, we were able to efficiently extract four homologues with various detergents while maintaining their oligomeric state. The ability to be solubilized by a wide range of detergents indicates that the proteins behave biochemically stable upon extraction from the membrane and will allow a broad screen of crystallization conditions, increasing our chances of obtaining well-diffracting crystals.

Subsequent to the successful identification of suitable detergents by FSEC, we performed large-scale purifications of *Alpo*1-eGFP, *Alpo*4-eGFP and *Alpo*6-eGFP. The GFP-tag allowed us to monitor the presence and monodispersity of our protein during every step of the purification protocol. For *Alpo*1-eGFP and *Alpo*4-eGFP this resulted in high yields of monodisperse and oligomeric protein. By contrast, the purification of *Alpo*6-eGFP resulted in low amounts of polydisperse protein. However, we were able to obtain a more symmetric, oligomeric peak by the addition of the low affinity agonist, glycine, during every step of purification. Still, purified *Alpo*6-eGFP appeared to be less monodisperse than the other *Alpo* constructs as shown by negative stain EM. FSEC-TS confirmed the beneficial effect of glycine on the thermal stability of *Alpo*6-GFP. Since GABA and taurine, did not exert these stabilizing effects, we could not ascribe the increased stability of the glycine-bound structure to a difference in inherent stability between an apo and open or desensitized channel conformation. However, this is not surprising since a similar discrepancy was observed for the stabilizing effects of different antagonists on the P2X receptor [35].

Over the last decade, it has been demonstrated that thermal stability of proteins is of great importance for structural studies [54–56]. Additionally, it has been shown that proteins derived from extremophilic organisms exhibit superior stability [22]. FSEC-TS [35] experiments indeed confirmed the superior thermal stability of *Alpo*1-eGFP, *Alpo*4-eGFP and *Alpo*6-eGFP. Remarkable high melting temperatures could be observed, all of them were in the range of 60 to 80°C. The melting temperature of purified *Alpo*6-eGFP for example is 77 ± 1°C, not only exceeding the melting temperatures of eukaryotic members of the CLR family [27,35,53,57], but also clearly higher than the melting temperature of GLIC, a prokaryotic homologue with a T_m_ of 52°C [58]. However, we have to consider the presence of eGFP in the intracellular loop. This can cause a stabilization of the receptors, which can consequently increase the observed melting temperatures. This might for example be the case for *Alpo*1-eGFP, since the purification of *Alpo*1-wt resulted in less symmetric oligomeric peaks. To exclude the effect of eGFP, FSEC-TS can be applied to *Alpo*-wt while monitoring the intrinsic tryptophan fluorescence.

The ultimate goal is obviously to determine the structure of wild type receptors. Therefore, *Alpo*1-wt, *Alpo*4-wt and *Alpo*6-wt were purified according to the same protocol used for the purification of the corresponding eGFP-tagged constructs. For *Alpo*6-wt, for example, the addition of glycine again appeared essential to obtain proper yields of monodisperse protein. This is a clear confirmation that glycine stabilizes both *Alpo*6-eGFP as well as *Alpo*6-wt. In case of *Alpo*4-wt, purification resulted in high yields of highly pure and homogeneous protein. The resulting SEC-profile closely resembled the SEC-profiles obtained by our group for ELIC, a prokaryotic CLR, which led to well-diffracting protein crystals and eventually to high-resolution crystal structures [12].

To further increase our chances for obtaining well-diffracting protein crystals, we decided to generate crystallization chaperones as they have been shown useful in elucidating the structure of other eukaryotic CLRs [14,17]. So far, Nanobodies were only generated against *Alpo*1-wt. However, in the future additional Nanobodies will be generated for other CLR homologues as well, especially for the most promising homologue *Alpo*4-wt. We were primarily interested in Nanobodies that bind with a high stoichiometric ratio (number of Nanobodies per *Alpo*1 oligomer) to maximally increase the available surface area for the formation of crystal contacts. By FSEC-analysis, we identified Nb9 as the Nanobody that provokes the most pronounced shift in retention volume and presumably thus binds with the highest stoichiometry. Additionally, we observed that Nb9 increases the resistance of *Alpo*1-eGFP to heating. These two characteristics, the high binding stoichiometry and the thermal stabilization, make Nb9 highly suitable as chaperone in future structural studies. The same protocol will be applied for the selection of Nanobodies targeting *Alpo*4-wt and *Alpo*6-wt.

Overall, we here report the successful functional and biochemical characterization of CLR homologues derived from the extremophilic organism *Alvinella pompejana*. We described the relevance of these homologues as models for human pLGICs by the high percentages of sequence identity and the identification of known neurotransmitters as agonists. Additionally, a thorough biochemical characterization indicated that three of the identified *Alpo* CLR homologues are suitable candidates for future crystallization trials as can be exemplified by three main findings: Firstly, the ability to be successfully extracted from the membrane by a wide variety of detergents. Secondly, their ability to be purified in a pure and oligomeric state. Thirdly, the identification of stabilizing additives. Together, these results pave the way for future functional and structural studies, possibly aiding in the further elucidation of the structure-function relationship for this class of clinically relevant ion channels.

## Supporting Information

S1 Fig Multiple sequence alignment. Multiple sequence alignment including full-length sequences of the putative cation-selective channels *Alpo*1-4 (blue), anion-selective channels *Alpo*5-7 (yellow), CLRs with known structures (pink) and human CLR subunits with high sequence identity to the identified homologues. The degree of amino acid conservation is displayed in shades of blue. Secondary structure elements, retrieved from the m5-HT_3_A R crystal structure (Hassaine G, Deluz C, Grasso L, Wyss R, Tol MB, Hovius R, et al. X-ray structure of the mouse serotonin 5-HT3 receptor. Nature. 2014;512: 276–281), are indicated above the alignment, α-helices and β-strands are colored in red and green, respectively.
(TIF)

S2 Fig The effect of ligands on the biochemical behavior of *Alpo*6. (A) SEC-profile derived from *Alpo*6-eGFP purified in the absence of ligand (blue). (B) SEC-profile derived from *Alpo*6-eGFP purified in the presence of glycine (green). (C) SEC-profile derived from *Alpo*6-eGFP purified in the presence of taurine (pink). (D) SEC-profile derived from *Alpo*6-eGFP purified in the presence of GABA (orange). (E) SEC-profile from *Alpo*6-wt purified in the presence of glycine. (F) Histogram displaying the relative fluorescence of the oligomeric peak height derived from FSEC-TS experiments on *Alpo*6-eGFP. *Alpo*6-eGFP incubated at 4°C (black) and at 65°C (blue) in the absence of ligands. *Alpo*6-eGFP incubated at 65°C in the presence of glycine (green), taurine (pink) and GABA (orange).
(TIF)

S3 Fig The identification of Nb9 as crystallization chaperone for *Alp*o1. (A) FSEC profiles from *Alpo*1-eGFP (red) and *Alpo*1-eGFP in complex with Nb9 (green). (B) Histogram displaying the relative fluorescence of the oligomeric peak height of *Alpo*1-eGFP incubated at 4°C (red), *Alpo*1-eGFP incubated at 65°C (orange) and *Alpo*1-eGFP in complex with Nb9 incubated at 65°C (green).
(TIF)

S1 Table Overview of the detergents and their concentrations used during solubilization and (fluorescence) size-exclusion chromatography ((F)SEC). (TIF)
