# Supplementary figures and images for: Functional and Biochemical Characterization of Alvinella pompejana Cys-Loop Receptor Homologues
Source: PLoS One. 2016 Mar 21;11(3):e0151183. doi: 10.1371/journal.pone.0151183 (PMC4801368; doi:10.1371/journal.pone.0151183)

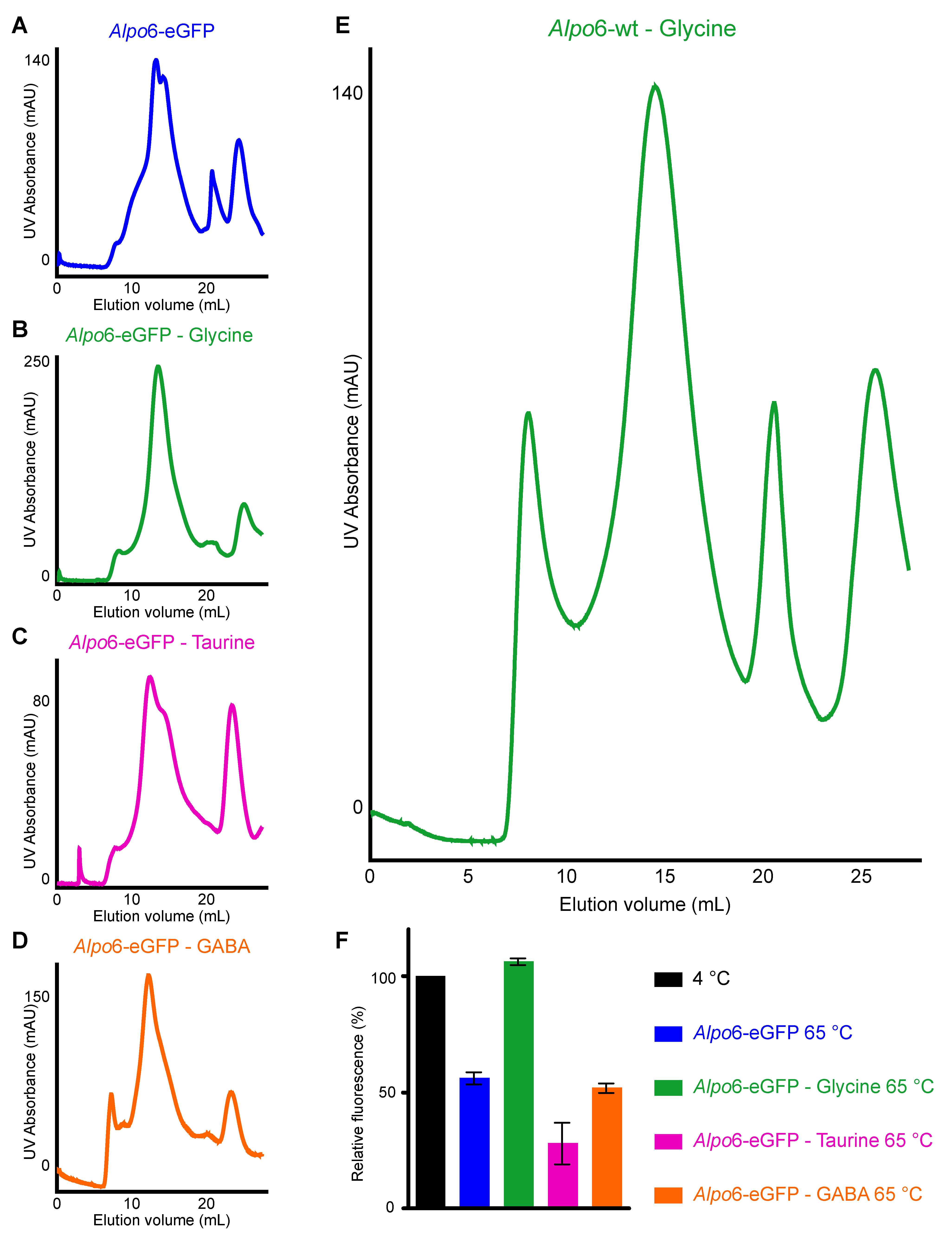

Supplement: S2 Fig — (A) SEC-profile derived from Alpo6-eGFP purified in the absence of ligand (blue). (B) SEC-profile derived from Alpo6-eGFP purified in the presence of glycine (green). (C) SEC-profile derived from Alpo6-eGFP purified in the presence of taurine (pink). (D) SEC-profile derived from Alpo6-eGFP purified in the presence of GABA (orange). (E) SEC-profile from Alpo6-wt purified in the presence of glycine. (F) Histogram displaying the relative fluorescence of the oligomeric peak height derived from FSEC-TS experiments on Alpo6-eGFP. Alpo6-eGFP incubated at 4°C (black) and at 65°C (blue) in the absence of ligands. Alpo6-eGFP incubated at 65°C in the presence of glycine (green), taurine (pink) and GABA (orange). (TIF) [file pone.0151183.s002.tif]

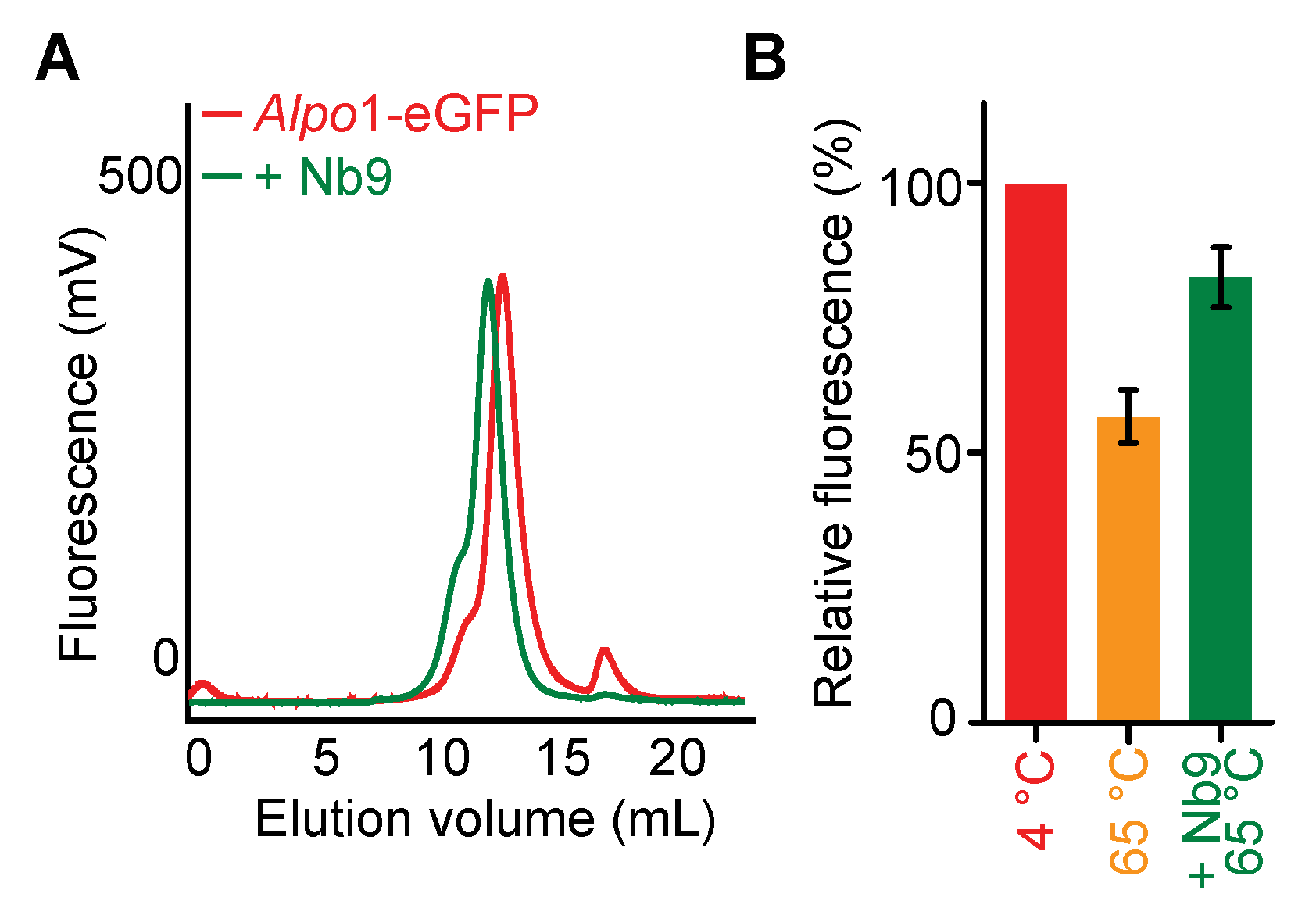

Supplement: S3 Fig — (A) FSEC profiles from Alpo1-eGFP (red) and Alpo1-eGFP in complex with Nb9 (green). (B) Histogram displaying the relative fluorescence of the oligomeric peak height of Alpo1-eGFP incubated at 4°C (red), Alpo1-eGFP incubated at 65°C (orange) and Alpo1-eGFP in complex with Nb9 incubated at 65°C (green). (TIF) [file pone.0151183.s003.tif]

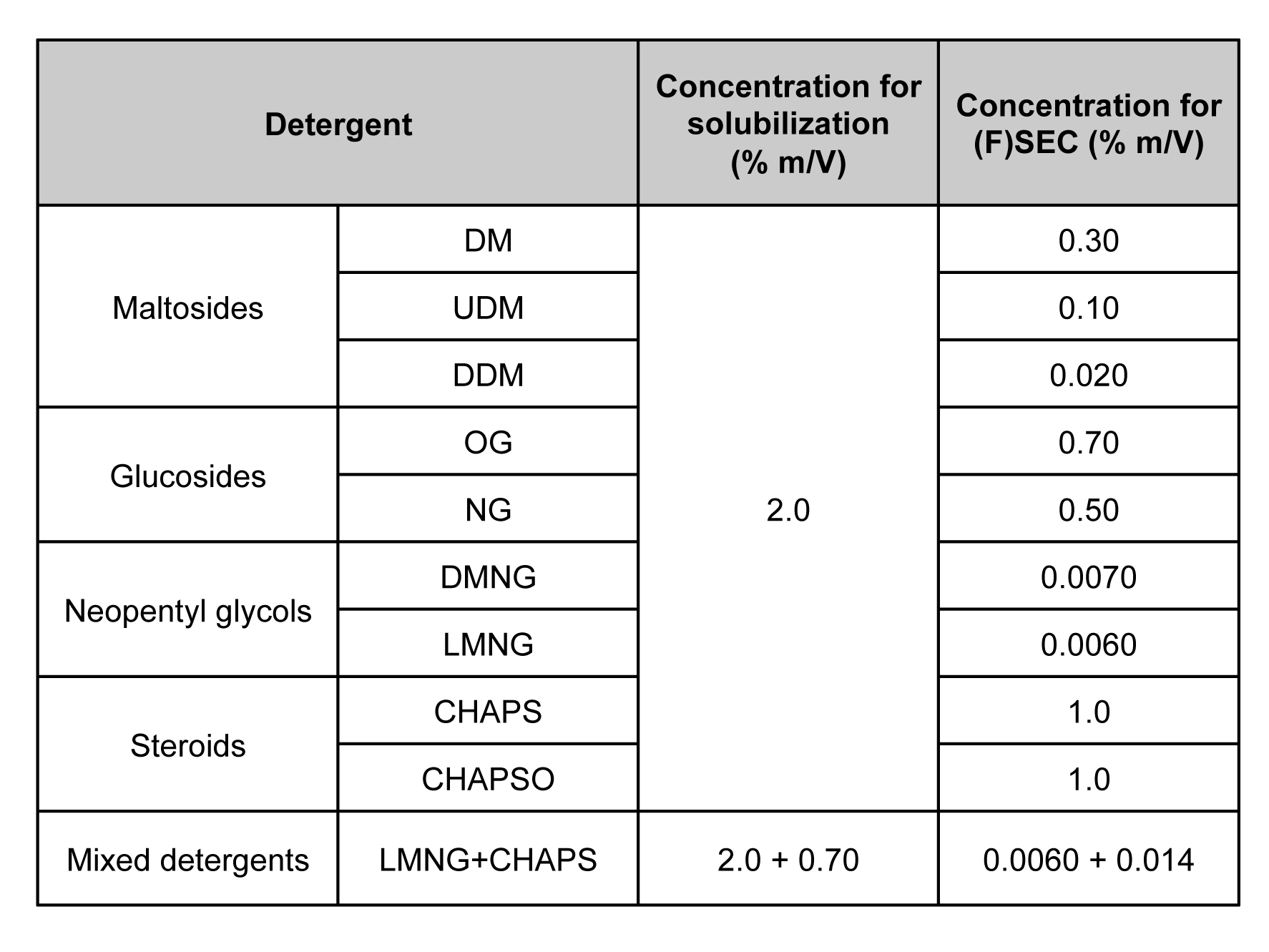

Supplement: S1 Table — (TIF) [file pone.0151183.s004.tif]
